# New Is Old, and Old Is New: Recent Advances in Antibiotic-Based, Antibiotic-Free and Ethnomedical Treatments against Methicillin-Resistant *Staphylococcus aureus* Wound Infections

**DOI:** 10.3390/ijms17050617

**Published:** 2016-04-25

**Authors:** Jian-Lin Dou, Yi-Wei Jiang, Jun-Qiu Xie, Xiao-Gang Zhang

**Affiliations:** 1 Institute of Pathogenic Biology, School of Basic Medical Sciences, Lanzhou University, Lanzhou 730000, China; doujl@lzu.edu.cn (J.-L.D.); xiejq@lzu.edu.cn (J.-Q.X.); 2 Spinal Surgery Department, Affiliated Hospital of Gansu University of Chinese Medicines, Lanzhou 730020, China; jiangyiwei_lz@163.com

**Keywords:** methicillin-resistant *Staphylococcus aureus* (MRSA), wound infection, biofilm, antibiotics, ethnomedicine

## Abstract

*Staphylococcus aureus* is the most common pathogen of wound infections. Thus far, methicillin-resistant *S. aureus* (MRSA) has become the major causative agent in wound infections, especially for nosocomial infections. MRSA infections are seldom eradicated by routine antimicrobial therapies. More concerning, some strains have become resistant to the newest antibiotics of last resort. Furthermore, horizontal transfer of a polymyxin resistance gene, *mcr-1*, has been identified in Enterobacteriaceae, by which resistance to the last group of antibiotics will likely spread rapidly. The worst-case scenario, “a return to the pre-antibiotic era”, is likely in sight. A perpetual goal for antibiotic research is the discovery of an antibiotic that lacks resistance potential, such as the recent discovery of teixobactin. However, when considering the issue from an ecological and evolutionary standpoint, it is evident that it is insufficient to solve the antibiotic dilemma through the use of antibiotics themselves. In this review, we summarized recent advances in antibiotic-based, antibiotic-free and ethnomedical treatments against MRSA wound infections to identify new clues to solve the antibiotic dilemma. One potential solution is to use ethnomedical drugs topically. Some ethnomedical drugs have been demonstrated to be effective antimicrobials against MRSA. A decline in antibiotic resistance can therefore be expected, as has been demonstrated when antibiotic-free treatments were used to limit the use of antibiotics. It is also anticipated that these drugs will have low resistance potential, although there is only minimal evidence to support this claim to date. More clinical trials and animal tests should be conducted on this topic.

## 1. Introduction

*Staphylococcus aureus* is the most common pathogen involved in skin and soft tissue infections and is the principal cause of surgical site infections (SSI) [1,2]. Wound infections often have negative impacts on patient outcomes, most commonly including a delay or deterioration of wound healing and potentially leading to sepsis. Furthermore, colonized staphylococcal cells are potential sources for cross-contamination, whereby *S. aureus* becomes a major nosocomial pathogen. Nosocomial infection is a major cause of surgical morbidity and mortality [3,4], and SSIs have a reported incidence rate of 2%–20% [5,6]. Evidence supports the use of *S*. *aureus* decolonization in surgical patients to prevent *S. aureus* infection, and this intervention has been associated with low rates of postoperative *S. aureus* infection. The staphylococcal carriage is most commonly eradicated by intranasal application of mupirocin either alone or in combination with antiseptic soaps or systemic antimicrobial agents [7]. However, the major cause of nosocomial infection is methicillin-resistant *S. aureus* (MRSA) [8,9,10], which is hard to eradicate [7] despite reports of some cases treated by warming therapy [4]. Furthermore, the efficacy of eradication in patients with community-associated MRSA has not been established, and the necessity of routine decolonization is not supported by data [7]. MRSA outbreaks have created a significant challenge for surgery and clinical practice in recent decades; the failure of traditional antimicrobial treatments has gradually become a worldwide problem [11,12,13], especially in the developing world [14]. Thus, effective therapeutic options to combat *S. aureus* infection, with an emphasis on MRSA, are urgently needed.

## 2. Antibiotics Developed to Combat MRSA

There are several therapeutic strategies, including clinical strategies and some still under development, to combat drug-resistant *S. aureus*: antibiotic-based treatments, alternative treatments, antibiotic-free treatments, immunotherapy, therapeutic vaccines and possible combinations of these options. [15,16,17]. Among these strategies, the use of antibiotics is effective and the most historically important. However, an unsettling trend has been witnessed in recent decades: increased antibiotic resistance and decreased antibiotic research and development [18,19,20]. From 1968 to 2003, only two novel classes of systemic antibiotics were developed: linezolid (2000) and daptomycin (2003) [19]. There are two primary contributors to this situation. Commercial interests with low profit motivations have failed to garner enthusiasm for antibiotic development because of their short-term use [13]. Second, the high level of difficulty in conducting clinical trials against drug-resistant strains dampens the hope of many candidates for final approval [18]. However, a new trend has emerged after the U.S. FDA (Food and Drug Administration)’s reboot of antibiotic development [20] involving an acceleration of drug approvals and documented antibiotic development. Some newly approved antibiotics (Table 1) are effective in preventing and controlling MRSA wound infections.

Antibiotic development is new, hard and time-consuming. However, a cost-effective and evidence-based strategy should be implemented to identify new therapeutic indications of clinically used drugs, *i.e.*, drug repurposing, rescue and repositioning [65]. This approach, although old, is both easy and time-saving. Ebselen is an organoselenium compound [66], and celecoxib is a marketed inhibitor of cyclooxygenase-2 [67]. Both exhibit demonstrated antimicrobial activity, especially for MRSA and vancomycin-resistant *S. aureus* (VRSA). Furthermore, ebselen has been shown to be remarkably active, to significantly reduce established staphylococcal biofilms and to act synergistically with traditional antimicrobials [66]. Simvastatin has been predicted to exhibit activity as a topical antimicrobial against skin bacterial infection due to its broad-spectrum (including MRSA) antibacterial, anti-staphylococcal biofilm, anti-inflammatory and wound healing activities [68]; furthermore, simvastatin can also synergistically act with traditional antimicrobials. Recently, tamoxifen, a selective estrogen receptor modulator widely used for the treatment of breast cancer, has been repositioned to enhance MRSA clearance by boosting neutrophil bactericidal capacity [69].

However, there is an intrinsic obstacle to antibiotic development: antibiotics are the only drugs for which widespread use decreases their utility, *i.e.*, “fighting back by microbes” [70]. There is almost no exception among the novel antibiotics (Table 1). A dilemma of antibiotics thus arises: antibiotics themselves are the cause of many healthcare-acquired infections (HCAI), further leading to “a return to the pre-antibiotic era”, as the World Health Organization has warned [71]. If this situation were to come to pass, once-curable infections would become fatal. Frighteningly, [72] horizontal transfer of a polymyxin resistance gene, *mcr-1*, has been identified in Enterobacteriaceae, and it is believed that this marks the beginning of the spread of resistance to the last group of antibiotics, leading to increased numbers of pan-drug-resistant strains. However, there is some hope. Teixobactin [73], discovered in a screen of uncultured bacteria, is a novel antibiotic that may not trigger the development of resistance. The biosynthetic gene cluster has been identified for future synthetic approaches. No resistant mutants of *S. aureus* were obtained by either serial passage at sub-MIC (minimum inhibitory concentration) levels of teixobactin or after culture on media with four-fold MIC of teixobactin. However, these results merely imply the rare occurrence of chromosomal mutation; as noted [72], a plasmid-mediated transfer mechanism contributes significantly to multi- and even pan-drug resistance. Furthermore, before it is introduced to clinical practice, drug resistance should be considered from an ecological and evolutionary view [74]. For example, a biofilm is a complex community with spatial and physiological heterogeneity in which the evolution of an exploitative interaction occurs [75,76]. Such evolution provokes marked changes in the symbiotic nature of heterogeneous strains/species and affects community function [75], e.g., antibiotic resistance. A previous study [77] revealed that clinically relevant resistance to the last class of antibiotics can be derived from competitive interactions between bacterial cells in a staphylococcal biofilm. Taken together, these data indicate that the concern of “a return to the pre-antibiotic era” has not been lifted. Alternative antibiotic-free treatments are therefore urgently required.

## 3. Topical Antibiotic-Free Treatments against MRSA

Although their single use rarely eradicates MRSA infections, topical antibiotic-free treatments can alter microbial burden, inhibit biofilm formation and disrupt formed biofilms. Because biofilm formation confers antibiotic resistance to bacteria [78], these treatments also play crucial roles in combating drug-resistant microbes through the inhibition and disruption of biofilms. Furthermore, these treatments improve wound healing through the removal of necrotic tissue, the alteration of the microbial burden, the reduction of local edema, and tissue repair and regeneration through wound contraction and remodeling, increasing angiogenesis and blood flow [79,80,81,82,83]. Among these effects, debridement is essential for successful wound care. Debridement has been shown [82] to effectively reduce the microbial burden in MRSA wound infections in several modalities, including traditional sharp debridement, hydrosurgery and plasma-mediated bipolar radiofrequency ablation (PBRA). Combinations of these antibiotic-free treatments are believed to be a promising approach to cure drug-resistant *S. aureus* wound infections. For example, debridement can improve the topical efficacy of species-specific bacteriophages against *S. aureus* biofilm-infected wounds by disrupting the matrix mass to aid the penetration of these bacteriophages [83].

The most common topical treatment in wound care is the use of a dressing. The wound dressing improves wound healing and tissue restoration; furthermore, modern wound dressings often exhibit excellent antimicrobial activity conferred by either non-antibiotics or antibiotics or a combination of the two [84]. Recently, an electrochemical scaffold (“e-scaffold”) made of conductive carbon fabric was proposed as an alternative antibiotic-free wound dressing to eliminate difficult-to-treat biofilms [85]. Low and constant concentrations of H_2_O_2_ are generated by the dressing to destroy biofilms through the electrochemical conversion of oxygen by applying an electric potential to the “e-scaffold”. Although the test strain was multidrug-resistant *Acinetobacter baumannii*, the dressing is expected to be used as a treatment against MRSA and MRSA-formed biofilms. Another antibiotic-free dressing is used to deliver human β-defensin 3 (hBD-3) by a bioengineered skin tissue; the dressing contains NIKS^hBD-3^ stably expressing hBD-3 after a NIKS human keratinocyte cell line was transfected *ex vivo* with a construct containing an epidermis-specific promoter driving hBD-3. Use of the dressing has been shown to result in a therapeutically relevant reduction in growth of an MRSA mutant (also a resistant to daptomycin and vancomycin) in an animal model of infected third-degree burn wounds [86].

Negative pressure wound therapy (NPWT) and NPWTi (NPWT with instillation) are complicated dressings to manage large complex injuries to soft tissue and have been shown to effectively reduce the infection rate of both chronic non-healing and acute contaminated open wounds [79,80,81]. Despite the lack of definitive conclusions concerning their use, partially due to the lack of large randomized controlled trials (RCTs), evidence from a small number of retrospective comparative cohorts demonstrates an association between the use of NPWT and a reduction in SSI [87]. Furthermore, clinical practices indicate their relevance in the prevention and control of MRSA infections [79,88,89]. Combined with antibiotics, NPWT successfully treated a pump pocket MRSA infection after a left ventricular assist device implantation [88] and a peri-prosthetic MRSA infection after incisional herniorrhaphy [89]. However, the results of a previous study demonstrate that NPWT reduces the effectiveness of local antibiotic depots, which is an adjunctive therapy for open wounds [79]. Large prospective RCTs of NPWT are therefore needed to support the current evidence that it is effective in treating MRSA with the adjunction of antibiotic therapy.

A previous case report indicated that acupuncture and moxibustion improved staphylococcal wound healing and resulted in full recovery with a partial withdrawal of antibiotics in a patient with a poor response to antibiotics, suggesting their complementary role in staphylococcal wound care [90]. Interestingly, larval therapy has been used as an alternative treatment for hard-to-heal chronic and infected wounds [91]. Some anti-MRSA factors have been identified in larval excretions/secretions [92]. Furthermore, larval therapy can eliminate *S. aureus* biofilms with the adjunction of antibiotic treatments [93]. These treatments provide clues to combat MRSA infection using natural means, on which ethnomedicine depends, for the long term.

## 4. Ethnomedical Treatments against MRSA

Antibiotics are gradually losing the war against microbes worldwide [11,12,13,70,71,72], and greater suffering can be observed in the developing world [14]. The desperate situation urges people to revisit the “old-fashioned” but still in use ethnomedicine, which roots from practical, empirical and “almost forgotten” knowledge about naturally derived materials. The resources are primarily based on botanical materials (herbs) but may include animal and mineral materials. Many active compounds have been identified from these materials; furthermore, some of them can be engineered and synthesized. In fact, natural products contribute significantly to modern healthcare. However, most ethnomedical treatments and remedies are yet to be verified, and more evidence is required to support their modern use. For example, acupuncture and moxibustion represent evidence-based complementary therapies for some diseases [94,95,96]. Although they have been reported to successfully treat MRSA infections [90], no evidence-based recommendations have been reported, nor are there ongoing clinical trials in PubMed, Cochrane Library and CenterWatch.

The unsuitability of natural product-centered paradigms in ethnomedical study is perhaps a responsible factor for the slow translation of ethnomedicine to evidence-based medicine. Ethnomedicinal materials may contain complex active compounds that result in a broad but vague spectrum of their ethnomedical indications due to pleiotropic effects. Additionally, the prescribed formulas may have different recipes to improve their therapeutic efficacy through altering the proportions of ingredients (in Chinese Traditional Medicine, the so-called “pattern differentiation and treatment determination, Bianzheng lunzhi”) [97]. “Treat as a whole” is the superior approach in the pharmacological study of those materials.

In accordance with the historical context and philosophy of their ethnomedical treatment, people in the developing world continue to use some naturally derived materials as topical wound healing agents, following the “treat it as a whole” approach. To facilitate audience reading, we have summarized in Table 2 some agents (either single or formula) that exhibit anti-staphylococcal activities. These agents exhibit complex activities on either planktonic microbes or biofilms or both. Their bactericidal effects are mediated by the inhibition of essential survival factors [98] or the microbial enzymatic activities [99,100] of the pathogens, by the antiseptics (e.g., H_2_O_2_ and methylglyoxal) that they contain [101], produce [102,103,104] and stabilize [105], and by harnessing physiological factors through active compounds, such as antimicrobial peptides [106] and alkaloids [107]. At the biofilm level, these agents target all formation stages, including the planktonic stage, initial adhesion to surfaces and sessile micro-colony formation. The agents inhibit the expression of key genes involved in biofilm formation [108], act as quorum sensing (QS) inhibitors [109,110], inhibit microbial adhesion by either impairing adherence ability of those biofilm-forming microbes [111] or by covering microbes and/or surfaces [78] and decreasing the produced biomass [112], thereby inhibiting formation and improving the eradication of biofilms.

Ethnomedical drugs may combat drug-resistant strains through their anti-biofilm activities because biofilm formation confers antibiotic resistance to bacteria [78]. For example, the medicinal plant *Duabanga grandiflora* has been demonstrated to inhibit MRSA biofilm formation through the reduction of cell-surface attachment and the attenuation of the level of penicillin-binding protein 2a (PBP2a) [143]. PBP2a, encoded by *mec A*, is a protein that confers β-lactam antibiotic resistance to *S. aureus*, thereby promoting the emergence of MRSA [144]. Furthermore, naturally derived materials may contain some inhibitors of multidrug efflux pumps [145], which are used to detoxify antibiotics by multi- and pan-resistant *S. aureus* [146]. Together, naturally derived materials can be used to reverse microbial antibiotic resistance and therefore aid in limiting the overuse of antibiotics. However, whether they themselves have a low resistance potential remains unclear. Indeed, “MRSA” is a medical term but not an ethnomedical one. The use of ethnomedicine remains insufficiently supported by evidence: ethnomedical drugs effectively treat MRSA infections with a low resistance potential.

## 5. A Feasible and Cost-Effective but Challenging Way: To Use Ethnomedical Drugs Topically

The topical use of ethnomedical drugs is not only an ethnomedical practice but a paradigm for ethnomedicine study. The complexity of their effects may decrease to a large extent while in topical use. A reverse proof is deduced from the selection between systemic and topical antibiotics for wound infection. Systemic antibiotics are often the putative preferred choice, perhaps because the antibiotic bioavailability is more stable than that of topical treatments when loci ischemia occurs [147]. Using ethnomedical drugs topically in wound dressings is therefore feasible and, given the ease of obtaining these drugs, cost-effective.

Based on naturally derived materials or natural products, several new wound dressings have been under development through either combination with other antimicrobial agents or when used alone [112]. For example, aloe vera inner gel has been demonstrated to exhibit antimicrobial activities against some Gram-negative (G^−^) bacteria and fungi [112]. Silver [148,149] and honey [101], both well-known, naturally derived antimicrobials, have also been used in the development of new wound dressings. Polymer films containing silver nanoparticles can confer an anti-MSSA (methicillin-sensitive strains of *Staphylococcus aureus*) effect on biological dressings [150]. Manuka honey synergistically enhanced the effectiveness of several antibiotics against MRSA and *Pseudomonas aeruginosa* [103] and can also exert positive effects on wound dressings when combined with silver, thereby lowering antimicrobial resistance and limiting the overuse of antibiotics [151]. Moreover, several herbs have been demonstrated to enhance silver nanoparticles (AgNPs) [152,153], and some of these herbs can also act as stabilizers of AgNPs [152]. Indeed, silver is generally accepted as an evidence-based topical wound antimicrobial that is used as a preparation of either a solution or nanoparticles in wound dressings [148,149]; however, silver is suggested to treat G^−^ bacteria more effectively, whereas mupirocin is the recommendation for MRSA wound infection [154].

A prerequisite of using ethnomedical drugs to combat MRSA infections more robustly than antibiotics is to make fewer mistakes in their use than were made in antibiotic use. A mistake that is still made is to make poor choices between systemic and topical administrations for wound infection. Systemic antibiotics are ideally never applied in topical use due to the risks of promoting both resistance and allergy [155], whereas topical antibiotics are not recommended for systemic use due to serious adverse effects [156]. Few topical antibiotics have been proven to be effective in clinical trials [156,157], although topical treatment with retapamulin and mupirocin is significantly more effective than systemic treatment with linezolid and vancomycin in eradicating MRSA in a murine superficial skin wound infection model [158]. Moreover, the topical use of antibiotics may result in systemic toxicity, allergy, wound healing delay and normal flora dysfunction [157,159]. Hence, it is crucial in wound care to rationally use systemic and topical antibiotics. Otherwise, overuse and misuse of antibiotics will drastically promote antibiotic resistance. One evidence-based suggestion is that topical antibiotic therapy might be an effective alternative to oral antibiotic therapy in treating diabetic patients with a mildly infected foot ulcer and might reduce the risk of selecting antimicrobial-resistant bacteria [159]. The overuse and misuse of topical antibiotics is perhaps due to an optimism bias that topical use rarely enhances the risk of antibiotic resistance. However, irrespective of whether the antibiotics are systemic or topical, widespread use is associated with the emergence of resistant bacterial strains [147]. In fact, topical antibiotics have long been known to promote the antibiotic resistance of *S. aureus* [155]. Clearly, the appropriate use of these agents remains a great challenge for antibiotic therapies for MRSA wound infections but a greater one for ethnomedical treatments. Nonetheless, there is still no clear definition of “systemic/topical” and “sensitive/resistant” in ethnomedicine.

To prevent the reoccurrence of a similar dilemma, the development and application of ethomedical drugs should avoid mistakes once made with antibiotics. Ethnomedical drugs should be divided into systemic and topical categories through evaluating their potential systemic adverse effects, allergies and toxicity, as well as local hypersensitivity and bio-absorbance, thereby avoiding misuse. To a large extent, the avoidance of antimicrobial misuse will result in a low resistance potential. However, the emergence of resistant mutants is the essential factor responsible for drug resistance. Thus, drug resistance should be monitored from the beginning of and throughout the development and application of ethnomedical drugs. Additionally, well-designed clinical trials and high-quality animal tests should be conducted because most of the ethnomedical studies on MRSA infection are conducted at the levels of case studies and *in vitro* assays (Table 2). The lack of evidence dampens the translation of ethnomedicine into evidence-based therapies.

## 6. Conclusions

Some newly developed antibiotics exhibit high effectiveness in combating MRSA infection, as do candidates under development. With a combination of debridement and modern wound dressings, these agents can successfully treat MRSA wound infections on the basis of limiting their usage. However, antibiotic resistance rapidly spreads, resulting in increasing numbers of multidrug- and even pan-drug-resistant strains. In addition to the development of novel antimicrobials and antibiotic-free treatments, the verification and validation of ethnomedical drugs is a feasible and cost-effective approach to address this issue. The topical use of ethnomedical drugs represents both the development of new wound dressings as well as a platform to study ethnomedical drugs in a “treat it as a whole” approach, which improves the translation of ethnomedicine into evidence-based practice. Naturally derived materials can therefore act as evidence-based drugs for modern medicine. The medical use of honey and silver is a successful paradigm. Despite the limited efficacy of their single use, their combined use in modern wound dressings contributes significantly to combating MRSA infections. Significantly, some naturally derived materials used in ethnomedicine may reverse the antibiotic resistance of MRSA through the attenuation of their PBP2a levels and the inhibition of their multidrug efflux pumps. The medicinal plant *Duabanga grandiflora* is therefore worthy of significant study. However, most ethnomedical materials are not well-studied, although they exhibit some relevance in combating MRSA infections based on *in vitro* assays. Furthermore, the tested strains were MSSA in some studies. Well-designed clinical trials and high-quality animal tests are therefore required in the future. Additionally, to avoid creating a similar crisis point in the future, the development and the application of ethnomedical drugs should learn the lessons of previous antibiotic treatment use.

## Figures and Tables

**Table 1:** Some U.S. Food and Drug Administration (FDA) approved antibiotics to combat methicillin-resistant *Staphylococcus aureus* (MRSA) infection [21].

Antibiotics	Indications	Therapeutic Relevance		Resistant Strains Reported	Month/Year Approved
		For MRSA Infections	For Wound Infections		
Oritavancin	For the treatment of acute bacterial skin and skin structure infections	[22,23]	[22,23]	Low potential [24]	August 2014
Sivextro	For the treatment of acute bacterial skin and skin structure infections	[25,26]	[27,28,29]	Low potential [25,30]	June 2014
Dalvance	For the treatment of acute bacterial skin and skin structure infections	[31,32]	[31,32]	VanA vancomycin-resistant enterococci [33]	May 2014
Teflaro	For the treatment of bacterial skin infections and bacterial pneumonia	[34,35,36]	[34,35,37]	A *S. aureus* [38]	October 2010
Telavancin	For the treatment of complicated skin and skin structure infections	[39,40,41]	[40,42]	VanA vancomycin-resistant enterococci [33]	September 2009; June 2013
Tigecycline	For the treatment of complicated skin and skin structure and intra-abdominal infections and bacterial pneumonia	[39,43,44,45,46]	[45,47,48,49]	*Staphylococcus* spp. [50], an MRSA [51], an *S. pneumoniae* [52]	June 2005
Daptomycin	For the treatment of complicated skin and skin structure infections	[39,43,45,53,54,55]	[45,53]	*Dermabacter hominis* [56], *Staphylococcus* spp. [45], a daptomycin non-susceptible *S. aureus* [57]	September 2003
Linezolid	For the treatment of infections, including pneumonia, infections of the skin and infections caused by a resistant bacterium (*Enterococcus faecium*)	[39,43,45,58,59]	[45,60]	Vancomycin-resistant *Enterococcus faecium*. [61]; MRSA [62,63,64]	April 2000

**Table 2:** Some ethnomedicinal materials with ethnomedical purposes for wound healing.

Regions	Resources of Materials	Effects		Note	References
		Anti-Biofilm	Anti-MRSA		
Bangladesh	*Bacopa monnieri* Linn. (Plantaginaceae)	Unknown	+	*In vitro* assays	[99]
Malaysia	*Cinnamomum* spp.	+[113]	+[71]	1. Better effects against MRSA than MSSA [71]	[71,113]
				2. *In vitro* assays	
	Leguminosae family	Unknown	+[114]	1. Anti-non-S.A. biofilm [115,116]	[114,115,116]
				2. *In vitro* assays	
China	Fructus Euodiae	Unknown	+	1. Active compounds: quinolone alkaloids	[107]
				2. *In vitro* assays	
	*Sanguisorba officinalis* L.	+	+	1. *In vitro* assays	[108]
				2. Inhibiting MRSA biofilm formation in an ica-dependent manner	
	*Toona sinensis* (A. Juss.) Roem. (TSL)	Unknown	+	1. Active compounds: sesquiterpenes	[117]
				2. *In vitro* assays	
Thailand	*Rhodomyrtus tomentosa* (Aiton) Hassk.	+[111]	+[118,119,120,121,122]	1. Active compound: rhodomyrtone	[111,118,119,120,121,122]
				2. Inhibiting microbial adherence ability to sustain surfaces	
	Herbal formulas	+[123]	+[124,125]	1. *In vitro* assays	[123,124,125,126]
				2. Ethnomedical purposes can be clues for their medical applications	
				3. Antagonistic interactions in combination with topical antiseptics	
Italy	Some medicinal plants	+	+	Ethnomedical purposes can be clues for their medical applications	[127]
Iran	*Malva sylvestris* L., *Solanum nigrum* L. and *Rosa damascene* Mill.	+[127]	+[127]	*In vitro* assays	[127,128]
African	*Ficus sansibarica Warb.* Subsp. *Sansibarica* (Moraceae)	+	+	*In vitro* assays	[129]
Ethiopia	*Guizotia* *schimperi* Sch. Bip. ex Walp.	Unknown	+	*In vitro* assays	[130]
Togo	*Balanites aegyptiaca* (L.) Delile (Balanitaceae)	Unknown	+	*In vitro* assays	[131]
African	*Aspilia africana* C. D Adams (Compositae)	Unknown	+[132]	Including animal tests	[133]
Australia	*Eremophila longifolia* (R. Br.) F. Muell	Unknown	+[134]	1. The first known Western scientific justification for the smoking ceremonies involving leaves of Eremophila longifolia	[135]
				2. *In vitro* assays	
Mediterranean	*Quercus cerris* L., Fagaceae	+	+	*In vitro* assays	[136]
Chilean	Some medicinal plants	Unknown	+	*In vitro* assays	[137]
Extensive	Tea tree	Unknown	+[138]	1. Clinical trial [138]	[138,139]
				2. Case study [139]	
North American	Lichens	Unknown	+	*In vitro* assays	[140]
Worldwide	Garlic	+[141]	+	*In vitro* assays	[141,142]

MSSA: Methicillin-sensitive *Staphylococcus aureus.*
